# Supplementary material for: Structural energetics of cold sensitivity
Source: Nature. 2026 Mar 25;653(8115):962–70. doi: 10.1038/s41586-026-10276-2 (PMC13190261; doi:10.1038/s41586-026-10276-2)
Supplement: Supplementary file 1 — Supplementary Tables 1–3 and Figs. 1–13. [file 41586_2026_10276_MOESM1_ESM.pdf]

nature portfolio

https://doi.org/10.1038/s41586-026-10276-2

Supplementary information

Structural energetics of cold sensitivity

In the format provided by the
authors and unedited

Structural energetics of cold sensitivity

Kevin Y. Choi [1,2,3,5], Xiaoxuan Lin [1,5], Yifan Cheng [1,4,6] and David Julius [2,6]

[1] Department of Biochemistry and Biophysics, University of California San Franscisco, San Francisco, CA, USA
[2] Department of Physiology, University of California San Franscisco, San Francisco, CA, USA
[3] Chemistry and Chemical Biology Graduate Program, University of California San Franscisco, San Francisco, CA, USA
[4] Howard Hughes Medical Institute
[5] These authors contributed equally to this work: Kevin Y. Choi and Xiaoxuan Lin
[6] address correspondence to yifan.cheng@ucsf.edu or david.julius@ucsf.edu

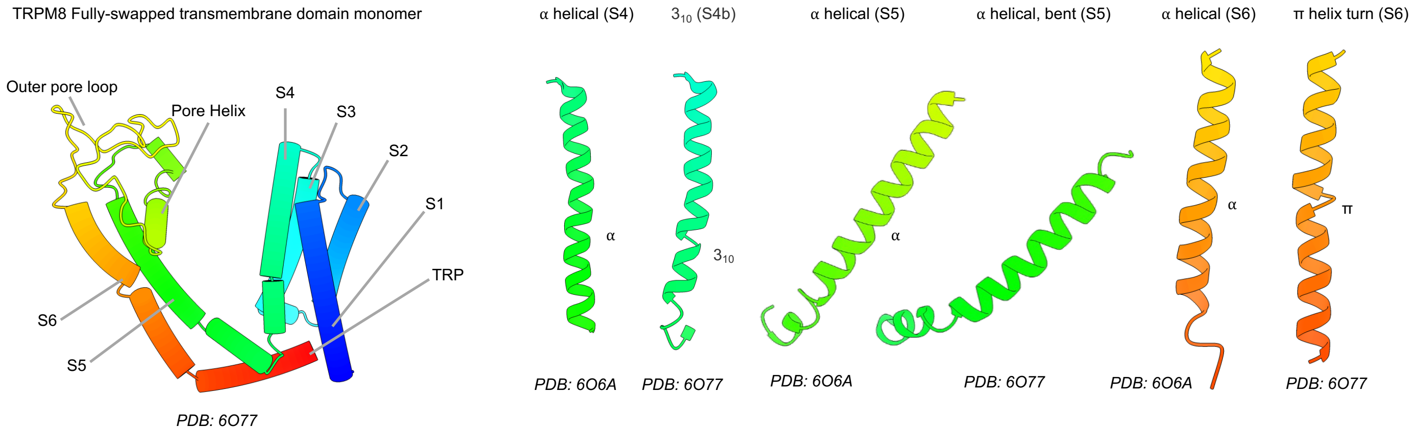

TRPM8 Structures in this study

EMD-ID (PDB)	Species	Sequence	Expression System	Preparation conditions	Ligands/Additives/Temperature	S4 region	S5 Helix	S6 Helix	Domain swap	Gating Residue (State)
EMD-71352 (9P7S)	*Parus major*	wild type	Expi293F	20 mM HEPES pH 7.4, 300 mM KCl, 1 mM DTT, 0.5 mM FFC8	5 mM EDTA, 4ºC	α helical	α helical	α helical	Fully swapped	Met968 + Phe969 (Closed)
EMD-71394 (9P90)	*Parus major*	wild type	Expi293F	20 mM HEPES pH 7.4, 300 mM KCl, 1 mM DTT, 0.5 mM FFC8	5 mM EDTA, 4ºC	3_{10} (S4b)	α helical, bent	π helix (Ser956 to Leu961)	Fully swapped	Val966 (Closed – Presumptive Desensitized)
EMD-71395 (9P91)	*Parus major*	wild type	Expi293F	20 mM HEPES pH 7.4, 300 mM KCl, 1 mM DTT, 0.5 mM FFC8	5 mM EDTA, 4ºC	3_{10} (S4b)	α helical, bent	α helical	Semi-swapped	Leu965 + Phe969 (Closed)
EMD-74126	*Parus major*	wild type	Expi293F	20 mM HEPES pH 7.4, 300 mM KCl, 1 mM DTT, 0.5 mM FFC8	5 mM EDTA, 4ºC	----	----	----	Undetermined	----
EMD-74127	*Parus major*	wild type	Expi293F	20 mM HEPES pH 7.4, 300 mM KCl, 1 mM DTT, 0.5 mM FFC8	5 mM EDTA, 4ºC	----	----	----	Undetermined	----
EMD-74037 (9ZCO)	*Parus major*	wild type	Expi293F	20 mM HEPES pH 7.4, 300 mM KCl, 1 mM DTT, 0.5 mM FFC8	5 mM CaCl2; 4ºC	3_{10} (S4b)	α helical, bent	α helical	Semi-swapped	Leu965 + Phe969 (Closed)
EMD-74036 (9ZCN)	*Parus major*	wild type	Expi293F	20 mM HEPES pH 7.4, 300 mM KCl, 1 mM DTT, 0.5 mM FFC8	5 mM CaCl2; 4ºC	α helical	α helical	α helical	Fully swapped	Met968 + Phe969 (Closed)
EMD-74123 (9ZEZ)	*Parus major*	wild type	Expi293F	20 mM HEPES pH 7.4, 300 mM KCl, 1 mM DTT, 0.5 mM FFC8	1 mM menthol, 5 mM EDTA, 4ºC	3_{10} (S4b)	α helical, bent	α helical	Semi-swapped	Phe969 (Open)
EMD-74039 (9ZCQ)	*Parus major*	wild type	Expi293F	20 mM HEPES pH 7.4, 300 mM KCl, 1 mM DTT, 0.5 mM FFC8	1 mM menthol, 5 mM EDTA, 4ºC	3_{10} (S4b)	α helical, bent	α helical	Semi-swapped	Phe969 (Closed)
EMD-74125	*Parus major*	wild type	Expi293F	20 mM HEPES pH 7.4, 300 mM KCl, 1 mM DTT, 0.5 mM FFC8	1 mM menthol, 5 mM EDTA, 4ºC	----	----	----	Fully swapped	Presumptive Desensitized
EMD-74038 (9ZCP)	*Parus major*	wild type	Expi293F	20 mM HEPES pH 7.4, 300 mM KCl, 1 mM DTT, 0.5 mM FFC8	1 mM menthol, 5 mM EDTA, 4ºC	α helical	α helical	α helical	Fully swapped	Met968 + Phe969 (Closed)
EMD-71444 (9PAR)	*Parus major*	wild type	Expi293F	20 mM TRIS pH 9.0, 150 mM NaCl, 5 mM $CaCl_2$, 0.0025% GDN	5 mM CaCl2; 4ºC	3_{10} (S4b)	α helical, bent	α helical	Semi-swapped	Phe969 (Open)
EMD-74040 (9ZCR)	*Parus major*	$pmTRPM8_{Human(V905-L938)}$	Expi293F	20 mM HEPES pH 7.4, 150 mM NaCl, 5 mM $CaCl_2$, 0.0025% GDN	5 mM CaCl2; 4ºC	3_{10} (S4b)	α helical, bent	α helical	Semi-swapped	Phe969 (Open)
EMD-71391 (9P8Y)	*Homo sapiens*	wild type	Expi293F	20 mM HEPES pH 7.4, 150 mM NaCl, 5 mM $CaCl_2$, 0.0025% GDN	5 mM CaCl2; 4ºC	α helical	α helical	α helical	Fully swapped	Met978 + Phe979 (Closed)
EMD-74041 (9ZCU)	*Homo sapiens*	hsTRPM8 V915Y	Expi293F	20 mM HEPES pH 7.4, 150 mM NaCl, 5 mM $CaCl_2$, 0.0025% GDN	5 mM CaCl2; 4ºC	α helical	α helical	α helical	Fully swapped	Met978 + Phe979 (Closed)
EMD-74128	*Homo sapiens*	hsTRPM8 V915Y	Expi293F	20 mM HEPES pH 7.4, 150 mM NaCl, 5 mM $CaCl_2$, 0.0025% GDN	5 mM CaCl2; 4ºC	----	----	----	Semi-swapped	----
EMD-74042 (9ZCV)	*Homo sapiens*	wild type	Expi293F	20 mM HEPES pH 7.4, 300 mM KCl, 1 mM DTT, 0.5 mM FFC8	1 mM EGTA, 1 mM EDTA; 4ºC	3_{10} (S4b)	α helical, bent	π helix (Ser966 to Leu971)	Fully swapped	Val976 (Closed – Presumptive Desensitized)
EMD-74124 (9ZF0)	*Homo sapiens*	wild type	Expi293F	20 mM HEPES pH 7.4, 300 mM KCl, 1 mM DTT, 0.5 mM FFC8	1 mM EGTA, 1 mM EDTA; 4ºC	3_{10} (S4b)	α helical, bent	α helical	Fully swapped	Phe979 (Open)
EMD-74129	*Homo sapiens*	wild type	Expi293F	20 mM HEPES pH 7.4, 300 mM KCl, 1 mM DTT, 0.5 mM FFC8	1 mM EGTA, 1 mM EDTA; 4ºC	----	----	----	Semi-swapped	----
EMD-71454 (9PB5)	*Homo sapiens*	wild type	Expi293F	20 mM HEPES pH 7.4, 300 mM KCl, 1 mM DTT, 0.5 mM FFC8	1 mM menthol, 1 mM EGTA, 1 mM EDTA; 4ºC	3_{10} (S4b)	α helical, bent	α helical	Fully swapped	Phe979 (Open)

Supplementary Information Table 1 | Summary of TRPM8 structures produced in this study.

This table summarizes all structures of TRPM8 determined in this study. Expi293F cells were used as the expression system for all proteins described in this study. EMDB / PDB codes, species, sequence (wild type or mutant), experimental conditions, and brief descriptions of structural features of transmembrane domains (depictured as shown above the table) are listed, including the configuration of S4 (a or 3_{10} helix), S5 (bent or not bent) and S6 (a or p) helices, domain architecture (full- or semi-swapped), gating residues and gating states (closed, open, and presumptive desensitized).

Published TRPM8 Structures and their Conditions

EMDB (PDB)	Species	Sequence	Expression System	Preparation conditions	Ligands/Additives/Temperature	S4 region	S5 Helix	S6 Helix	Domain Architecture	Gating Residues/states
6BPQ	*Ficedula albicollis*	F535A, Y538D, Y539D	HEK293S GnTi-	20 mM Tris pH 8, 150 mM NaCl, 0.06-0.1 % digitonin, 1 mM InsP6, 2 mM MgCl2 and 2 mM DTT	4ºC	α helical	α helical	α helical	Fully swapped	Leu973 (Closed)
6NR2	*Ficedula albicollis*	F535A, Y538D, Y539D	HEK293S GnTi-	20 mM Tris pH 8, 150 mM NaCl, 0.06-0.1 % digitonin, 1 mM InsP6, 2 mM MgCl2 and 2 mM DTT	1 mM diC8-PIP2 and 200 µM WS-12; 4ºC	α helical	α helical	α helical	Fully swapped	Met977 + Phe978 (Closed)
6NR3	*Ficedula albicollis*	F535A, Y538D, Y539D, A805G	HEK293S GnTi-	20 mM Tris pH 8, 150 mM NaCl, 0.06-0.1 % digitonin, 1 mM InsP6, 2 mM MgCl2 and 2 mM DTT	1 mM diC8-PIP2, 200 µM icilin, and 1 mM CaCl2; 4ºC	3_{10} (S4b)	α helical, bent	α helical	Fully swapped	Met977 (Closed)
6NR4	*Ficedula albicollis*	F535A, Y538D, Y539D, A805G	HEK293S GnTi-	20 mM Tris pH 8, 150 mM NaCl, 0.06-0.1 % digitonin, 1 mM InsP6, 2 mM MgCl2 and 2 mM DTT	1 mM diC8-PIP2, 200 µM icilin, and 1 mM CaCl2; 4ºC	α helical	α helical	α helical	Fully swapped	Met977 (Closed)
6O6A	*Parus major*	ΔN1-20	HEK293S GnTi-	20 mM HEPES, pH 7.4, 150 mM NaCl, PMAL-C8	2 mM EGTA, cholesteryl hemisuccinate (CHS); 4ºC	α helical	α helical	α helical	Fully swapped	Met977 + Phe978 (Closed)
6O6R	*Parus major*	ΔN1-20	HEK293S GnTi-	20 mM HEPES, pH 7.4, 150 mM NaCl, PMAL-C8	500 µM AMTB hydrochloride, cholesteryl hemisuccinate (CHS); 4ºC	α helical	α helical	α helical	Fully swapped	Met977 + Phe978 (Closed)
6O72	*Parus major*	ΔN1-20	HEK293S GnTi-	20 mM HEPES, pH 7.4, 150 mM NaCl, PMAL-C8	250 µM TC-I 2014, cholesteryl hemisuccinate (CHS); 4ºC	α helical	α helical	α helical	Fully swapped	Met977 + Phe978 (Closed)
6O77	*Parus major*	ΔN1-20	HEK293S GnTi-	20 mM HEPES, pH 7.4, 150 mM NaCl, PMAL-C8	2 mM $CaCl_2$, cholesteryl hemisuccinate (CHS); 4ºC	3_{10} (S4b)	α helical, bent	π helix (Ser956 to Leu961)	Fully swapped	Val966 (Closed – Presumptive Desensitized)
7WRA	*Mus musculus*	wild type	HEK293F	20 mM Tris-HCl, pH 8.0, 150 mM NaCl, 0.0025% LMNG, and 0.0005% CHS	2 mM EGTA, cholesteryl hemisuccinate (CHS); 4ºC	3_{10} (S4b)	α helical, bent	π helix (Ser966 to Leu971)	Fully swapped	Val976 (Closed – Presumptive Desensitized)
7WRB	*Mus musculus*	wild type	HEK293F	20 mM Tris-HCl, pH 8.0, 150 mM NaCl, 0.0025% LMNG, and 0.0005% CHS	cholesteryl hemisuccinate (CHS); 4ºC	3_{10} (S4b)	α helical, bent	π helix (Ser966 to Leu971)	Fully swapped	Val976 (Closed – Presumptive Desensitized)
7WRC	*Mus musculus*	wild type	HEK293F	20 mM Tris-HCl, pH 8.0, 150 mM NaCl, 0.0025% LMNG, and 0.0005% CHS	200 µM icilin, 0.5 mM CaCl2, 0.5 mM diC8-PIP2, cholesteryl hemisuccinate (CHS); 4ºC	3_{10} (S4b)	α helical, bent	π helix (Ser966 to Leu971)	Fully swapped	Val976 (Closed – Presumptive Desensitized)
7WRD	*Mus musculus*	wild type	HEK293F	20 mM Tris-HCl, pH 8.0, 150 mM NaCl, 0.0025% LMNG, and 0.0005% CHS	200 µM icilin, 0.5 mM CaCl2, cholesteryl hemisuccinate (CHS); 4ºC	3_{10} (S4b)	α helical, bent	π helix (Ser966 to Leu971)	Fully swapped	Val976 (Closed – Presumptive Desensitized)
7WRE	*Mus musculus*	wild type	HEK293F	20 mM Tris-HCl, pH 8.0, 150 mM NaCl	200 µM icilin, 0.5 mM CaCl2, cholesteryl hemisuccinate (CHS), POPC/POPE/POPG; 4ºC	3_{10} (S4b)	α helical, bent	π helix (Ser966 to Leu971)	Fully swapped	Val976 (Closed – Presumptive Desensitized)
7WRF	*Mus musculus*	wild type	HEK293F	20 mM Tris-HCl, pH 8.0, 150 mM NaCl	200 µM icilin, 0.5 mM CaCl2, 0.5 mM diC8-PIP2, cholesteryl hemisuccinate (CHS), POPC/POPE/POPG; 4ºC	3_{10} (S4b)	α helical, bent	π helix (Ser966 to Leu971)	Fully swapped	Val976 (Closed – Presumptive Desensitized)
8E4L	*Mus musculus*	wild type	HEK293F	20 mM Tris-HCl pH 8, 150 mM NaCl, 0.02% GDN	1 mM diC8-PIP2, 1 mM C3, and 500 µM AITC; 20ºC	3_{10} (S4b)	α helical, bent	π helix (Y963 to Asn968)	Fully swapped	Val976 (Open)
8E4M	*Mus musculus*	wild type	HEK293F	20 mM Tris-HCl pH 8, 150 mM NaCl, 0.02% GDN	1 mM diC8-PIP2, 1 mM C3; 20ºC	3_{10} (S4b)	α helical, bent	α helical	Fully swapped	Phe979 + Val983 (Closed)
8E4N	*Mus musculus*	wild type	HEK293F	20 mM Tris-HCl pH 8, 150 mM NaCl, 0.02% GDN, 5 mM EDTA	1 mM diC8-PIP2; 20ºC	3_{10} (S4b)	α helical, bent	α helical	Fully swapped	Phe979 + Val983 (Closed)
8E4O	*Mus musculus*	wild type	HEK293F	20 mM Tris-HCl pH 8, 150 mM NaCl, 0.02% GDN, 5 mM EDTA	20ºC	3_{10} (S4b)	α helical, bent	α helical	Fully swapped	Phe979 + Val983 (Closed)
8E4P	*Mus musculus*	wild type	HEK293F	20 mM Tris-HCl pH 8, 150 mM NaCl, 0.02% GDN, 0.004% CHS, 5 mM EDTA	cholesteryl hemisuccinate (CHS); 20ºC	α helical	α helical	α helical	Fully swapped	Met978 + Phe979 (Closed)
8E4Q	*Ficedula albicollis*	F535A, Y538D, Y539D	HEK293S GnTi-	20 mM Tris pH 8, 150 mM NaCl, 0.06-0.1 % digitonin	1 mM diC8-PIP2; 4ºC	α helical	α helical	α helical	Fully swapped	Met977 + Phe978 (Closed)
8BDC	*Homo sapiens*	wild type	SF9	20 mM HEPES pH 7.4, 150 mM NaCl, 2 mM Na-EGTA, 0.025 mM LMNG, 0.005 mM CHS, and 100 µM Tris[2-carboxyethyl]phosphine	cholesteryl hemisuccinate (CHS); 4ºC	α helical	α helical	α helical	Fully swapped	Met978 + Phe979 (Closed)
9B6D	*Mus musculus*	wild type	HEK293F	20 mM tris-HCl (pH 8), 150 mM NaCl, 0.005% LMNG, and 0.001% CHS	5 mM EDTA, cholesteryl hemisuccinate (CHS); 20ºC	3_{10} (S4b)	α helical, bent	π helix (Ser966 to Leu971)	Fully swapped	Met978 + Phe979 (Closed)
9B6E	*Mus musculus*	wild type	HEK293F	20 mM tris-HCl (pH 8), 150 mM NaCl, 0.005% LMNG, and 0.001% CHS	300 µM TC-I 2014, 5 mM EDTA, cholesteryl hemisuccinate (CHS); 20ºC	3_{10} (S4b)	α helical, bent	π helix (Ser966 to Leu971)	Fully swapped	Val976 (Closed – Presumptive Desensitized)
9B6F	*Mus musculus*	wild type	HEK293F	20 mM tris-HCl (pH 8), 150 mM NaCl, 0.005% LMNG, and 0.001% CHS	400 µM AMG2850, 5 mM EDTA, cholesteryl hemisuccinate (CHS); 20ºC	3_{10} (S4b)	α helical, bent	π helix (Ser966 to Leu971)	Fully swapped	Val976 (Closed – Presumptive Desensitized)
9B6G	*Mus musculus*	wild type	HEK293F	20 mM tris-HCl (pH 8), 150 mM NaCl, 0.005% LMNG, and 0.001% CHS	500 µM AMTB, 5 mM EDTA, cholesteryl hemisuccinate (CHS); 20ºC	3_{10} (S4b)	α helical, bent	π helix (Ser966 to Leu971)	Fully swapped	Val976 (Closed – Presumptive Desensitized)
9B6H	*Mus musculus*	wild type	HEK293F	20 mM tris-HCl (pH 8), 150 mM NaCl, 0.005% LMNG, and 0.001% CHS	300 µM TC-I 2014, 1 mM C3, 5 mM EDTA, cholesteryl hemisuccinate (CHS); 20ºC	3_{10} (S4b)	α helical, bent	π helix (Ser966 to Leu971)	Fully swapped	Val976 (Closed – Presumptive Desensitized)
9B6I	*Parus major*	wild type	HEK293S GnTi-	20 mM tris-HCl (pH 8), 150 mM NaCl, 0.05% LMNG, 0.01% CHS, and 1 mM CaCl2	300 µM TC-I 2014, 1 mM CaCl2; 20ºC	3_{10} (S4b)	α helical, bent	π helix (Ser972 to Leu977)	Fully swapped	Val982 (Closed – Presumptive Desensitized)
9B6J	*Mus musculus*	wild type	HEK293F	20 mM tris-HCl (pH 8), 150 mM NaCl, and 0.02% GDN	1 mM diC8-PIP2, 1 mM CaCl2; 20ºC	3_{10} (S4b)	α helical, bent	α helical	Fully swapped	Phe979 + Val983 (Closed)
9B6K	*Mus musculus*	wild type	HEK293F	20 mM tris-HCl (pH 8), 150 mM NaCl, and 0.02% GDN	1 mM CaCl2; 20ºC	α helical	α helical, bent	π helix (Ser966 to Leu971)	Fully swapped	Val976 (Closed – Presumptive Desensitized)

Supplementary Information Table 2 | Summary of published TRPM8 structures in the PDB.

This table summarizes all available structures of TRPM8 deposited in the PDB. PDB codes, species, sequence (wild type or mutant), experimental conditions, and brief descriptions of structural features of transmembrane domains (depictured as shown above the table) are listed, including the configuration of S4 (a or 3_{10} helix), S5 (bent or not bent) and S6 (a or p) helices, domain architecture (full- or semi-swapped), gating residues and gating states (closed, open, and presumptive desensitized).

Dataset	pmTRPM8, 4 °C	pmTRPM8, 22 °C	pmTRPM8, 30 °C	pmTRPM8, 37 °C	hTRPM8, 4 °C	hTRPM8, 22 °C	hTRPM8, 30 °C	hTRPM8, 37 °C	pmTRPM8, 22 °C, extract in menthol	pmTRPM8, 22, °C, add'n menthol	hTRPM8, 22 °C, extract in menthol	hTRPM8, 22, °C, add'n menthol
HDX reaction details	20 mM HEPES, 150 mM NaCl, 0.005% GDN, pD_{read} 7.0								20 mM HEPES, 150 mM NaCl, 1 mM menthol 0.005% GDN, pD_{read} 7.0			
HDX time course (*: no replicate. Times in parentheses: times in pD_{read} 7.0, 22 °C after correcting for the k_{chem} difference)	263 s (30 s), 2627 s (5 min), 26270 s (50 min), 3d58min (500 min)*	30 s, 5 min, 50 min, 500 min	19 s (30 s), 186 s (5 min), 1865 s (50 min), 18649 (500 min)	10 s (30 s), 97 s (5 min), 973 s (50 min), 9730 (500 min)	26s (3 s)*, 263 s (30 s), 831 s (95 s)*, 2627 s (5 min), 8307 s (16 min)*, 26270 s (50 min), 3d58min (500 min)*	30 s, 5 min, 50 min, 500 min, 30 h	19 s (30 s), 186 s (5 min), 1865 s (50 min), 18649 (500 min)	10 s (30 s), 97 s (5 min), 973 s (50 min), 9730 (500 min)	30 s, 5 min, 50 min, 500 min	30 s, 5 min, 50 min, 500 min	30 s, 5 min, 50 min, 500 min	30 s, 5 min, 50 min, 500 min
Controls	Non-deuterated control; in-exchange control; maximally labeled control											
In- and back-exchange, mean/IQR	In-exchange: 0.6% / 1.6%; back-exchange: 25% / 13%				In-exchange: 0.3% / 1.7%; back-exchange: 24% / 14%				In-exchange: 0.6% / 1.6%; back-exchange: 25% / 13%		In-exchange: 0.3% / 1.7%; back-exchange: 24% / 14%	
No. of peptides	1007				1068				1007		1068	
Sequence coverage	94.6%				95.8%				94.6%		95.8%	
Average peptide length/redundancy	10.3/9.5				10.2/9.9				10.3/9.5		10.2/9.9	
Replicates	3	3	3	3	3	3	3	3	3	2	3	2
Repeatability (mean of the SD)	1.9%/0.15 Da	2.4%/0.19 Da	2.3%/0.17 Da	2.29%/0.18 Da	1.8%/0.14 Da	2.3%/0.18 Da	2.5%/0.20 Da	2.8%/0.22 Da	2.3% / 0.15 Da (TMD peptide only)	1.7% / 0.11 Da (TMD peptide only, SD of Δ%D and Δ#D between duplicates)	2.5% / 0.2 Da (TMD peptide only)	1.6% / 0.13 Da (TMD peptide only, SD of Δ%D and Δ#D between duplicates)
Confidence Interval (95%)	22°C vs 4°C: 0.38 Da<br>30°C vs 22°C: 0.41 Da<br>37°C vs 30°C: 0.40 Da				22°C vs 4°C: 0.36 Da<br>30°C vs 22°C: 0.43 Da<br>37°C vs 30°C: 0.47 Da				0.36 Da (vs Apo 22 °C)	N/A	0.41 Da (vs Apo 22 °C)	N/A

Supplementary Information Table 3 | Biochemical and statistical details for HDX-MS

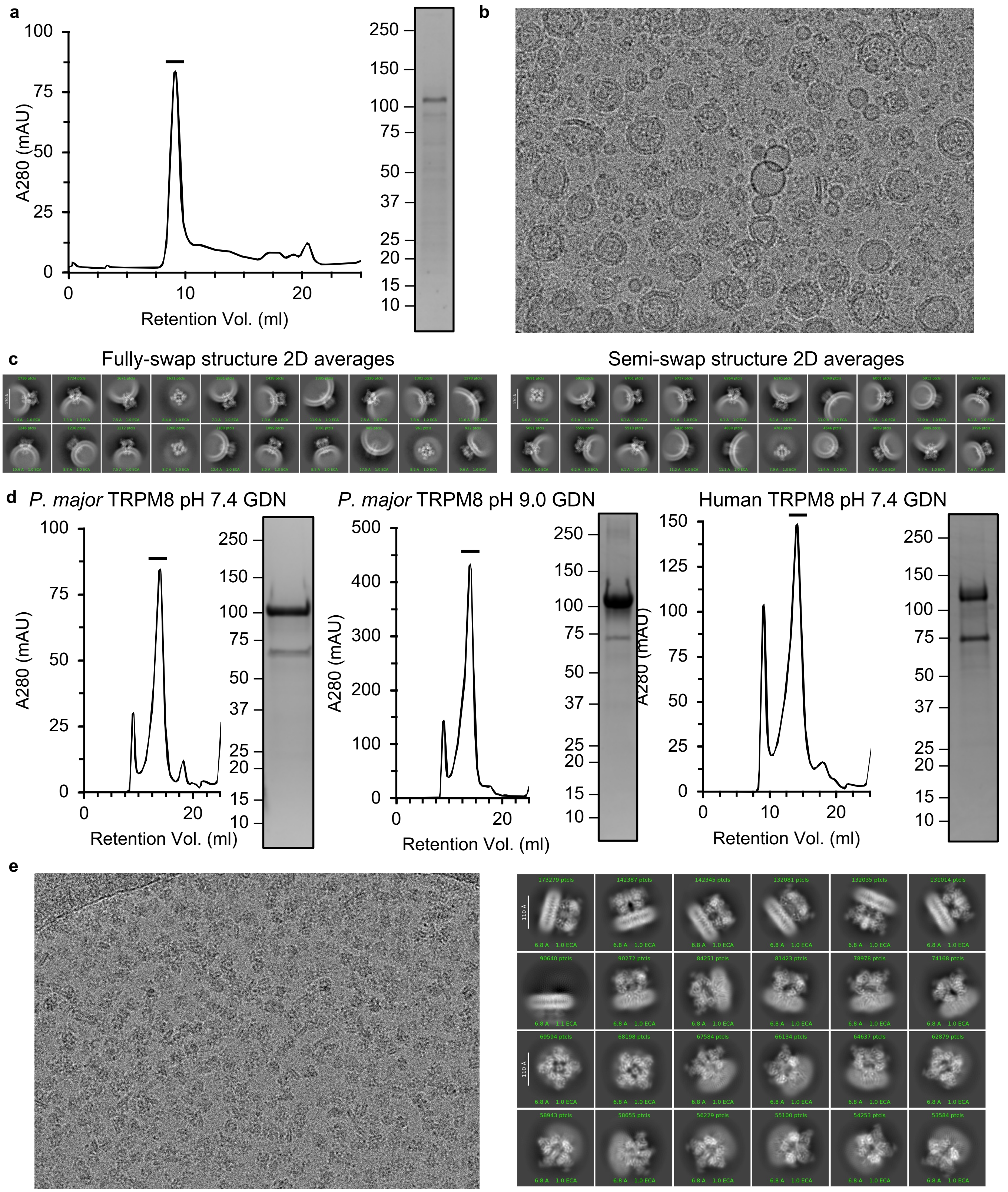

Supplementary Information Fig. 1 | Preparation and cryo-EM of TRPM8. (a) Representative size-exclusion chromatogram of TRPM8 in cell membrane vesicles. Black bar denotes representative region analyzed by Coomassie-stained SDS-PAGE gel. (b) Representative micrograph of TRPM8-containing vesicles. (c) Representative 2D class averages of TRPM8 in membrane vesicles from particles classified as fully- or semi-swapped channels as indicated, in each case showing a wide spread of membrane curvatures. (d) Purification of *pm*TRPM8 or *hs*TRPM8 in GDN. (e) Representative micrograph and 2D class averages of GDN-purified *pm*TRPM8 at high pH.

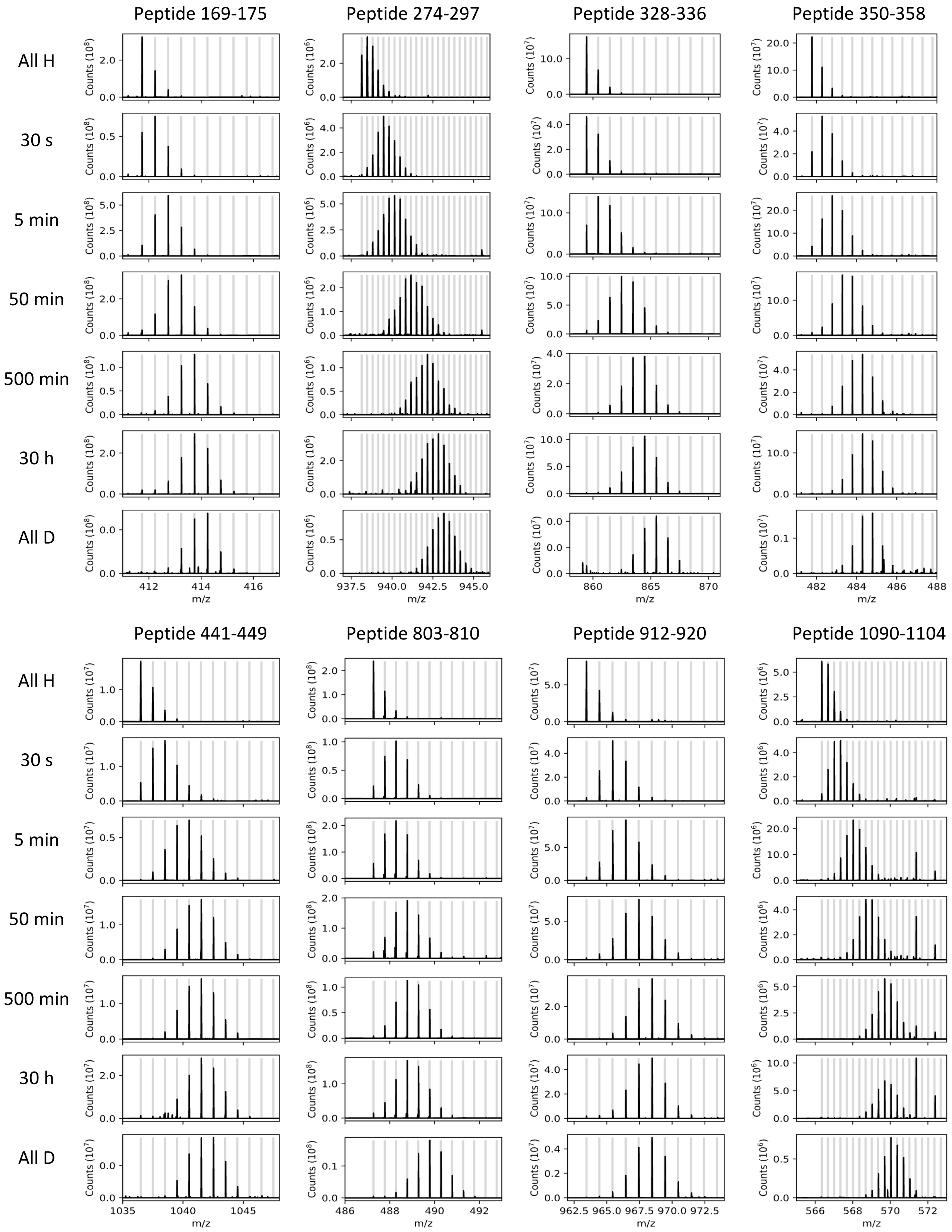

Supplementary Information Fig. 2 | Unimodal mass spectra for example hsTRPM8 peptides. Single mass envelopes show continuous increases in m/z over exchange time, consistent with exchange via EX2 mechanism. Gray horizontal bars indicate theoretical m/z values for the corresponding isotopes.

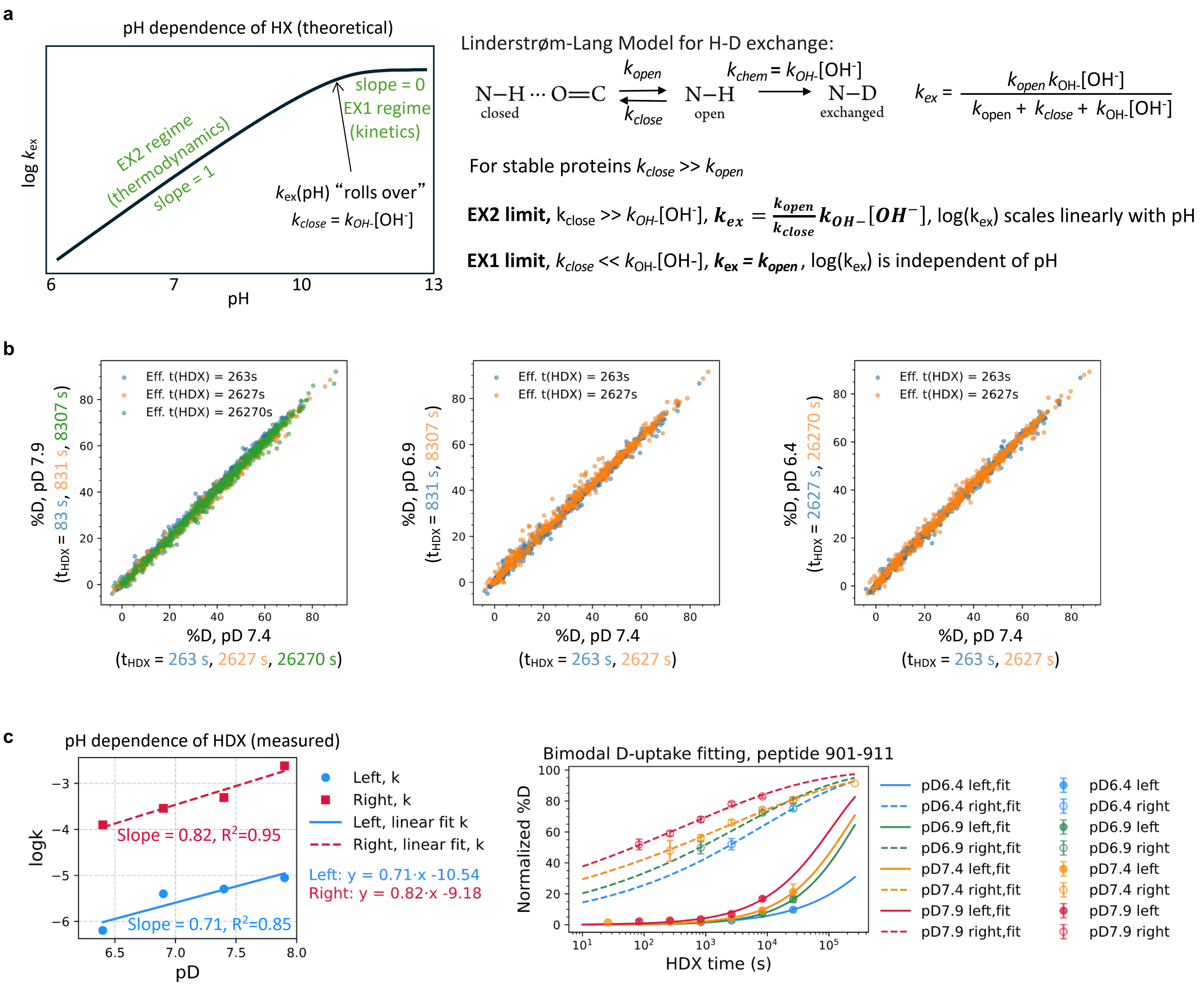

Supplementary Information Fig. 3 | pH dependence of HDX for TRPM8 demonstrates exchange via EX2 mechanism. (a) HX formalism demonstrating pH dependence of HX rates. Figure adapted from S. W. Englander & N. R. Kallenbach, 1984 and S. S. Jaswal & A. D. Miranker, 2007. (b) Comparison of deuteration levels for all unimodal peptides in hsTRPM8 labeled at 4 °C across different pD conditions (pD 6.4, 6.9, 7.4, 7.9). Variations in %Deuteration can be attributed solely to the effect of pD on k_{chem}, consistent with HDX occurring via EX2 kinetics. Effective t(HDX) are HDX times corrected to pD 7.4 based on k_{chem}. (c) Measured pD dependence of $\log(k_{ex})$ for the left and right mass envelopes of the bimodal peptide 901-911 (left) and the uptake curves with fitting using a stretched exponential method (right).

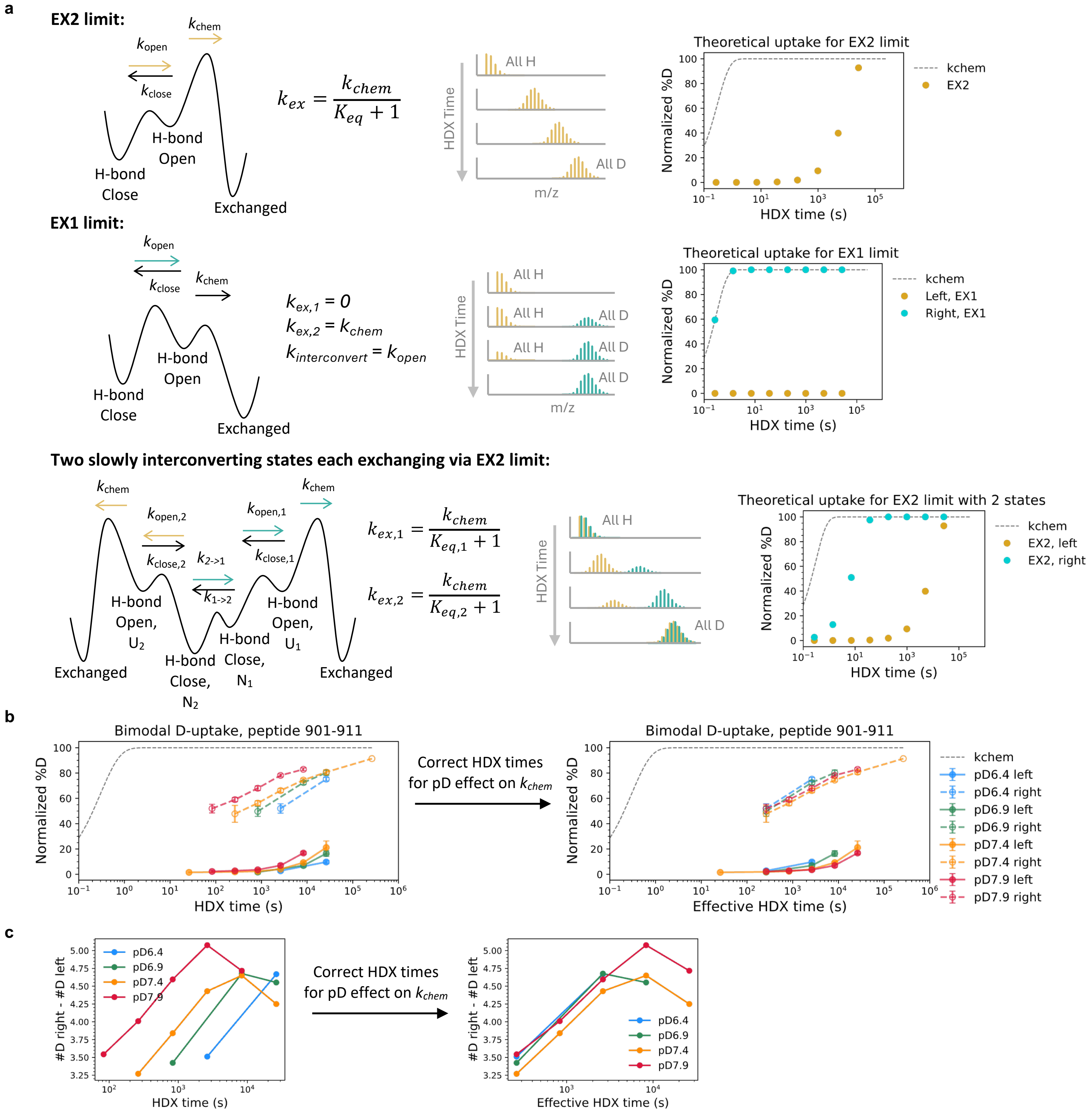

Supplementary Information Fig. 4 | Mass envelope shifts and pD-dependence of HDX for TRPM8 demonstrates exchange via EX2 mechanism. (a) Energy diagrams of HDX reactions with predicted mass spectra and uptake curves. Exchange via EX2 regime is identified by continuous increase in m/z for a single isotopic envelope over exchange time. In EX1 limit, the rate of reforming the hydrogen bond (k_{close}) is much slower than k_{chem}; therefore, every opening event results in exchange. The signature spectra of EX1 kinetics is a decrease in the amplitude of the lighter envelope and a commensurate increase in the heavier amplitude over time without a shift in m/z when $t_{HDX}>>1/k_{chem}$. Isotopic envelopes resulted from the presence of two slowly interconverting, structurally distinct populations also show bimodal mass spectra, but both envelopes show partial deuteration at rates slower than k_{chem}, reflecting the two populations each exchanging in EX2 regime. (b) Uptake curves for an example peptide showing bimodality in TRPM8. Both mass envelopes exchange with deuterium much slower than k_{chem}, with exchange rates scale with k_{chem}, demonstrating the presence of two structurally distinct populations, each exchanging via EX2 mechanism. (c) The difference in the number of deuteration between the two mass envelopes changes over HDX time and scales with k_{chem}, inconsistent with mixed EX1 in which a peptide contains some residues exchanging in EX1 and others exchanging in EX2.

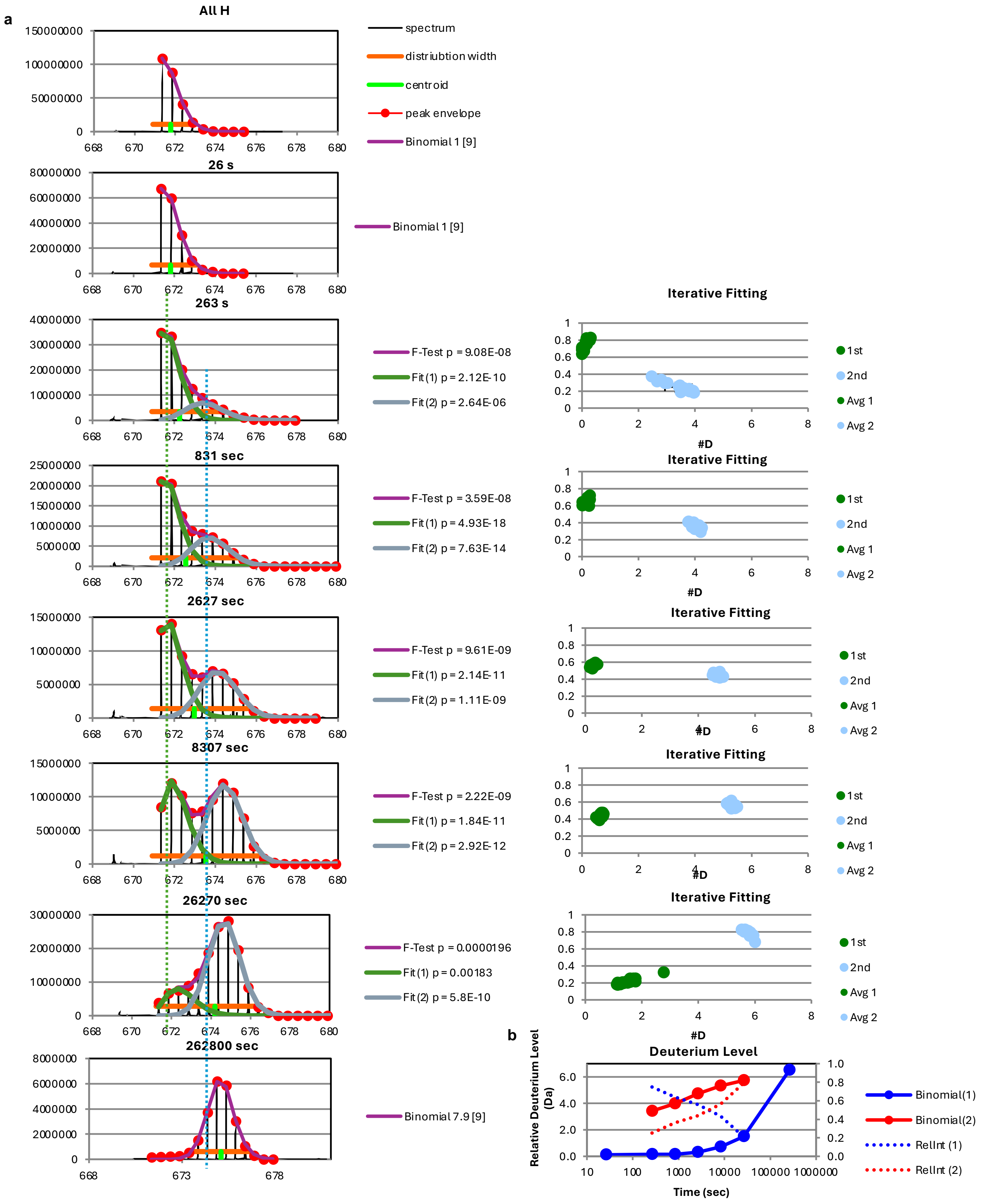

Supplementary Information Fig. 5 | Bimodal fitting of raw mass spectra for the pore helix peptide (residues 901-911) of hsTRPM8 labeled at pD7.4 and 4°C. Double-binomial fitting was performed with HX-Express3 (Tuttle *et al.*, 2025). Fitting was repeated with 20 iterations using a resampling approach in which random noise (±30% of each isotope peak intensity) was introduced in each iteration. The green and blue dotted lines on the mass spectra denote the centroids of the left and right mass envelopes from the fitting results of the spectra at t_{HDX}=263 s, to provide visual guidance for the m/z shift of both mass envelops over labeling time.

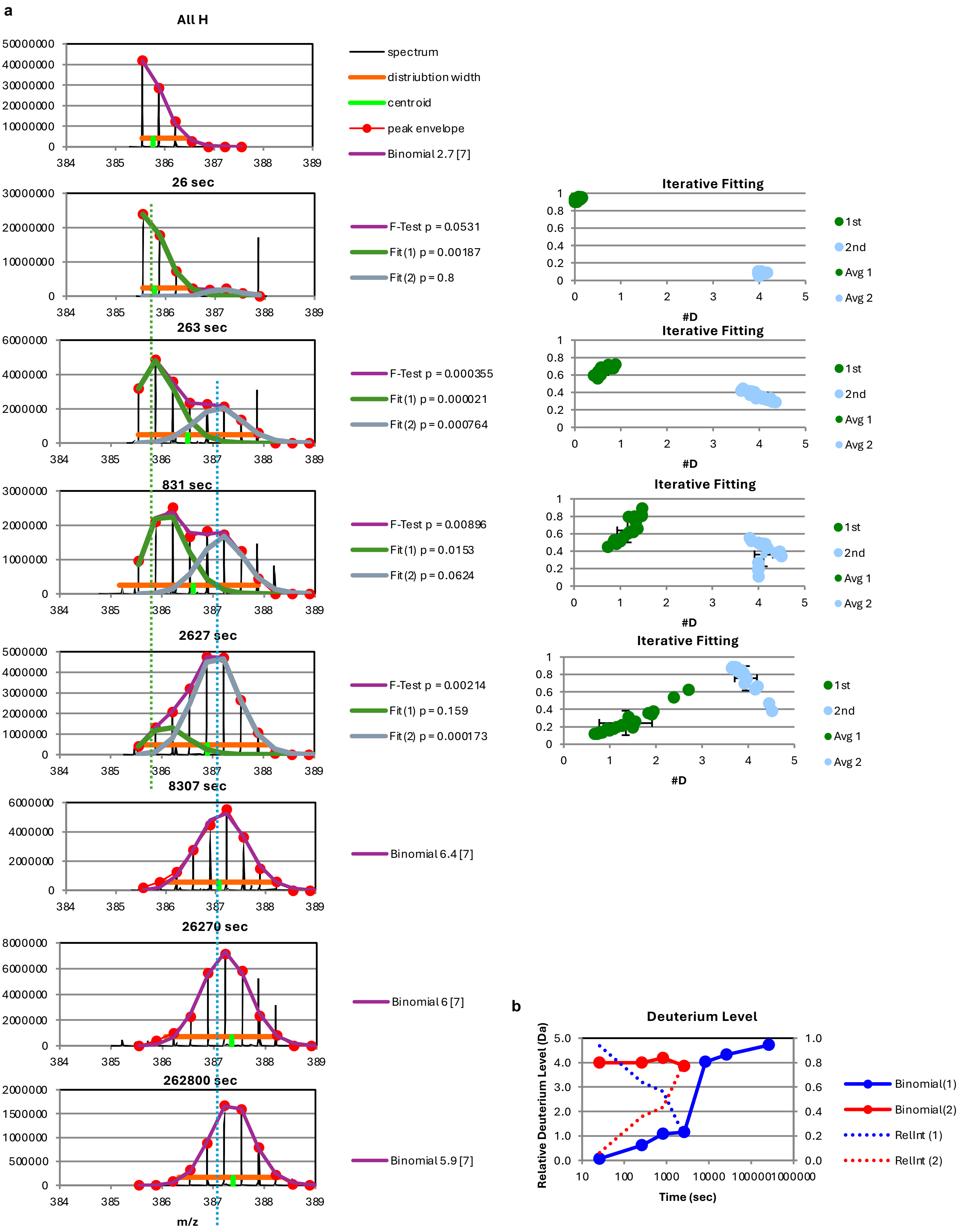

Supplementary Information Fig. 6 | Bimodal fitting of raw mass spectra for the TRP helix peptide (residues 992-999) of hsTRPM8 labeled at pD7.4 and 4°C. Double-binomial fitting was performed with HX-Express3 (Tuttle *et al.*, 2025). Fitting was repeated with 20 iterations using a resampling approach in which random noise (±30% of each isotope peak intensity) was introduced in each iteration. The green and blue dotted lines on the mass spectra denote the centroids of the left and right mass envelopes from the fitting results of the spectra at t_{HDX}=26 s and 263 s, respectively, to provide visual guidance for the m/z shift of both mass envelops over labeling time.

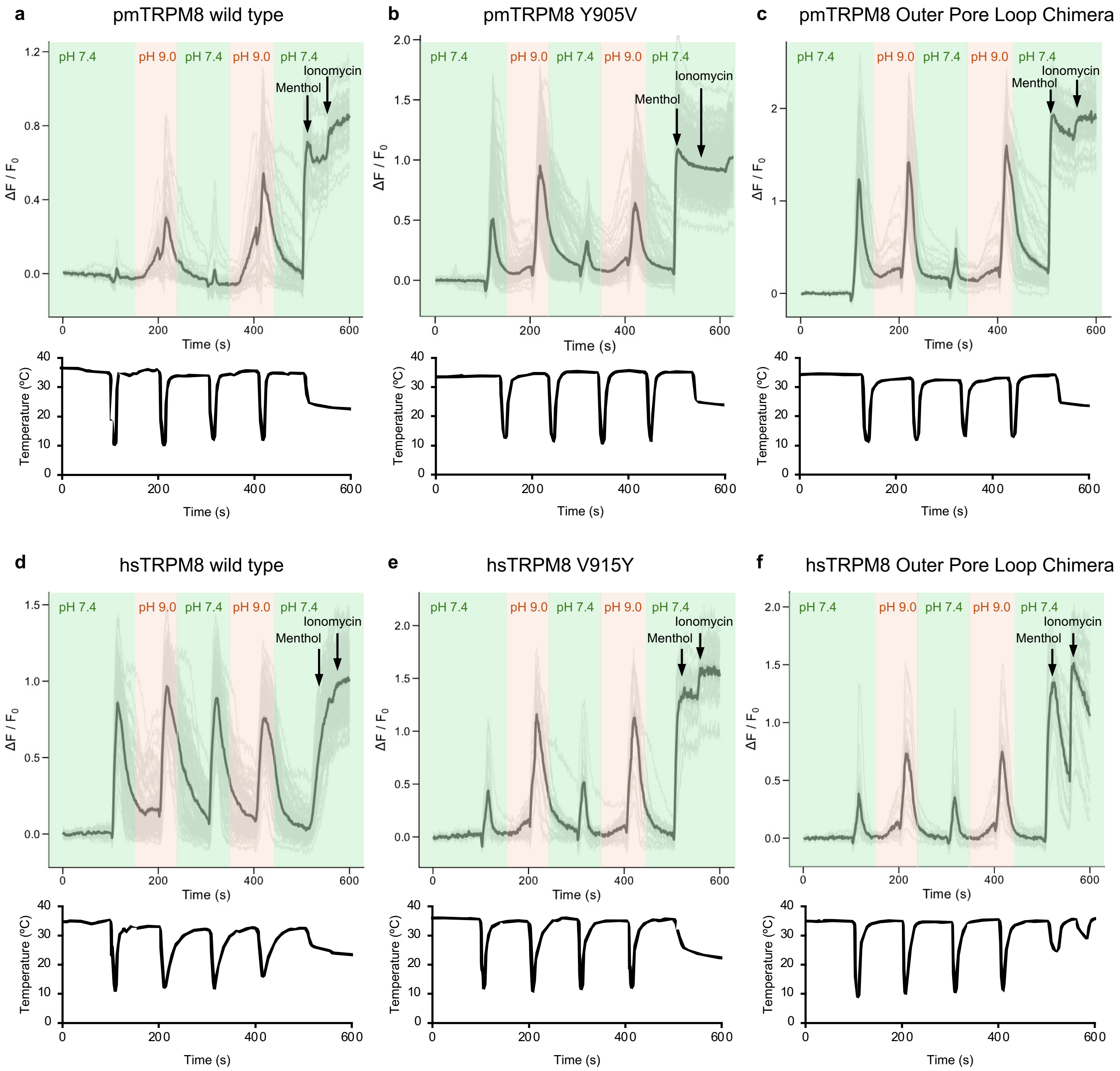

Supplementary Information Fig. 7 | Cold response of avian or human TRPM8. Representative calcium imaging traces obtained from HEK293T cells. Bolded traces represent the average response obtained from all measured cells, and light traces represent responses of individual cells. (a) *pm*TRPM8 wild type (n = 40), (b) *pm*TRPM8 Y905V (n = 336), (c) *pm*TRPM8 "humanized" outer pore loop chimera (n = 259), (d) *hs*TRPM8 wild type (n = 79), (e) *hs*TRPM8 V915Y (n = 86), and (f) *hs*TRPM8 "avianized" outer pore loop chimera (n = 71). Calcium responses were measured with cell-permeant, ratiometric dye Fura-2-AM. Temperature ramps are shown below each trace. Responses to cold or menthol (100 μM) were normalized to maximum calcium signal following application of 10 μM ionomycin. For figure 4, the first two cold responses were quantified.

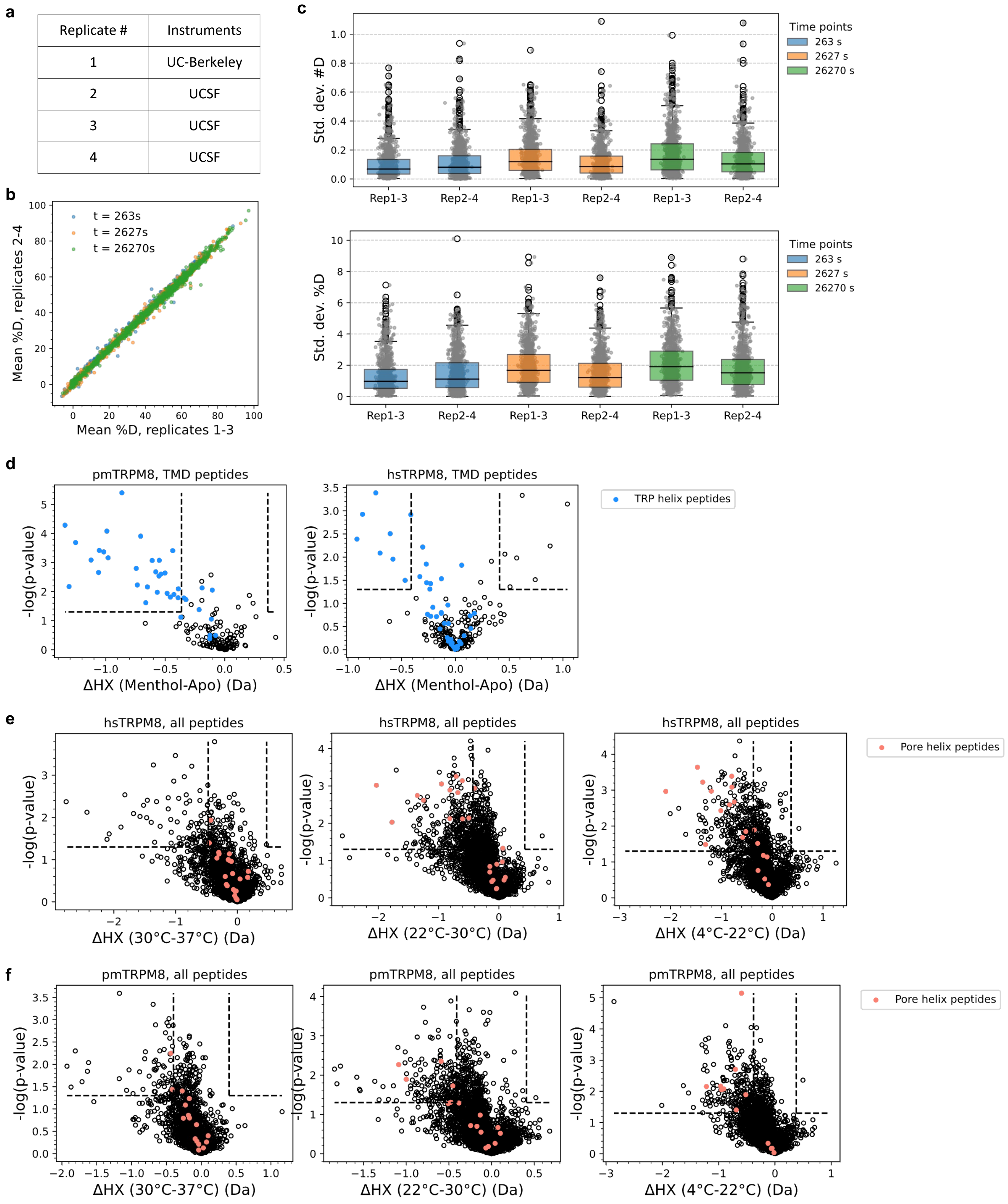

Supplementary Information Fig. 8 | HDX-MS data repeatability and statistics. (a-c) LC-MS instrument precision was evaluated by comparing the mean deuteration levels (b) and standard deviations (c) for all peptides at three HDX times for replicates 1-3 (containing a mixture of datasets analyzed using two different LC-MS instruments) versus replicates 2-4 (containing datasets analyzed with the same LC-MS instrument). (d-f) Volcano plot analysis of HDX for pmTRPM8 and hsTRPM8 in response to menthol binding (d) and temperature changes (e-f). A hybrid statistical analysis is employed (Hageman and Weis, 2019). Each data point represents one time point of a peptide. The horizontal axis shows the difference between the observed mean peptide masses ($\Delta\overline{HX}$). The vertical axis shows Welch's t-test p-values. The dashed horizontal lines denote p-value significance limits defined at α = 0.05. The dashed vertical lines denote the $\Delta\overline{HX}$ significance threshold corresponding to α = 0.05.

a *P. Major* TRPM8 determined in cell vesicles – cold adapted in the absence of calcium – closed/semi-swapped – PDB: 9P91 (EMD-71395)

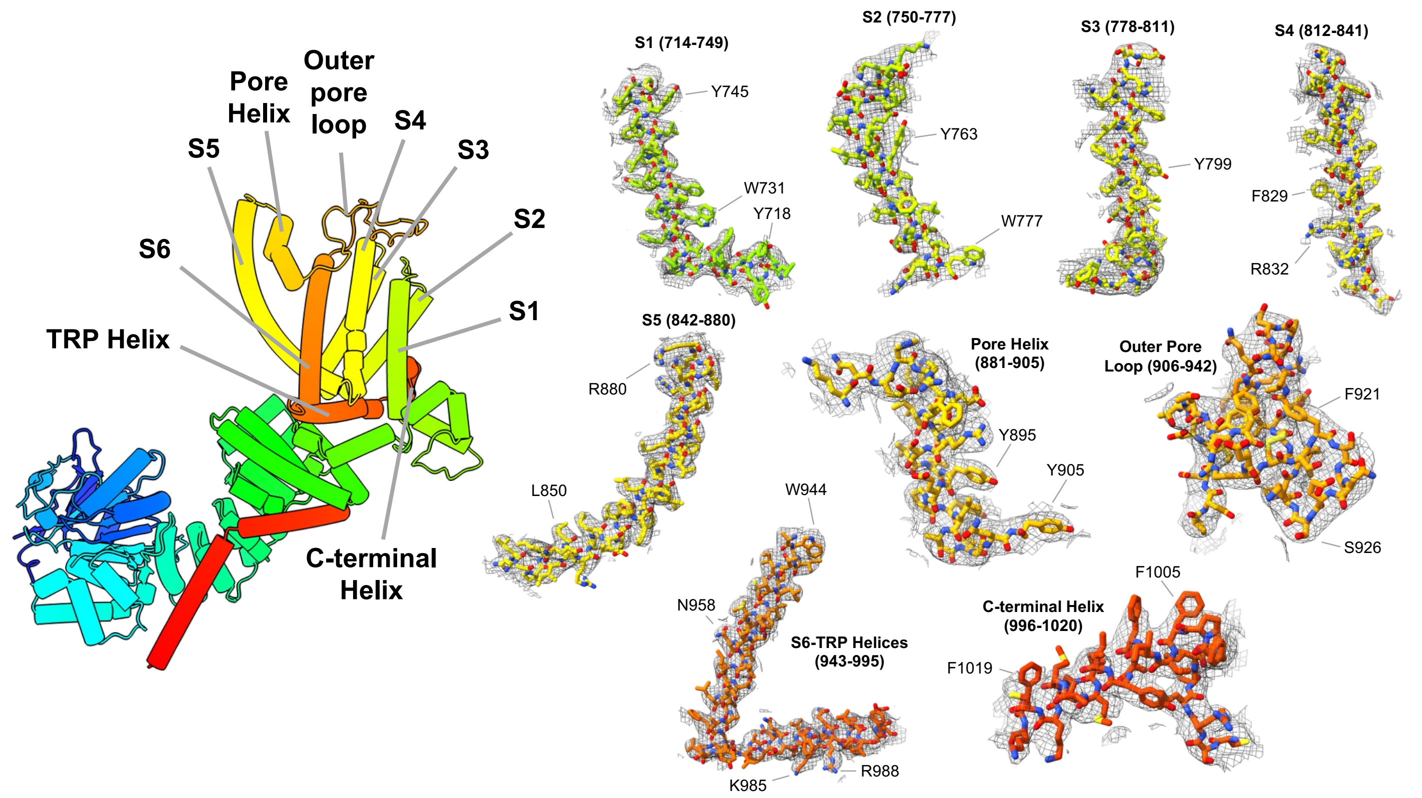

b *P. Major* TRPM8 determined in cell vesicles – cold adapted in the absence of calcium – desensitized/fully-swapped – PDB: 9P90 (EMD-71394)

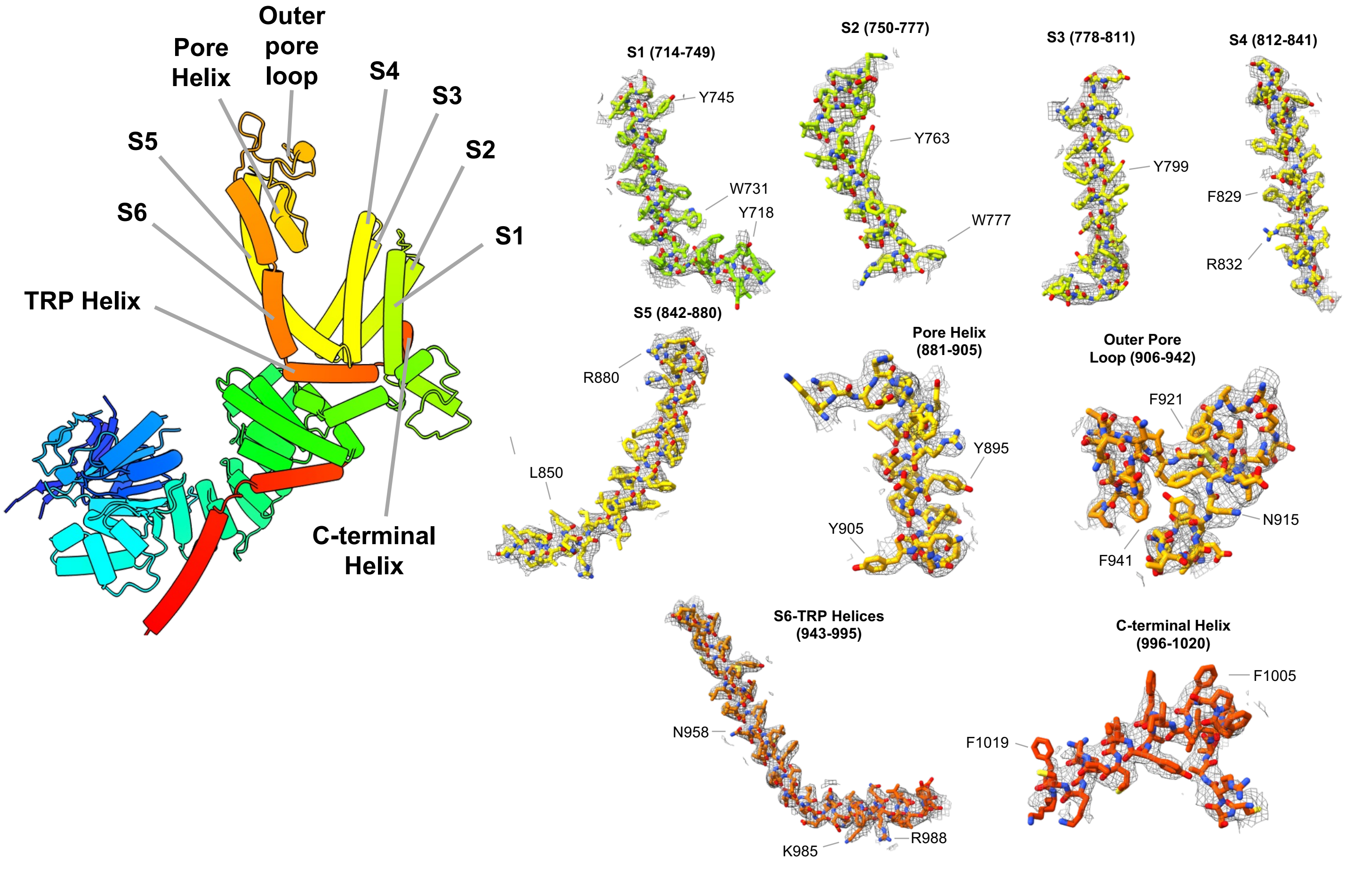

Supplementary Information Fig. 9 | Model and density representations. Zoned densities of transmembrane structure elements modelled in PDBs included in this study.

c *P. Major* TRPM8 determined in cell vesicles – cold adapted in the absence of calcium – closed/full-swapped – PDB: 9P7S (EMD-71352)

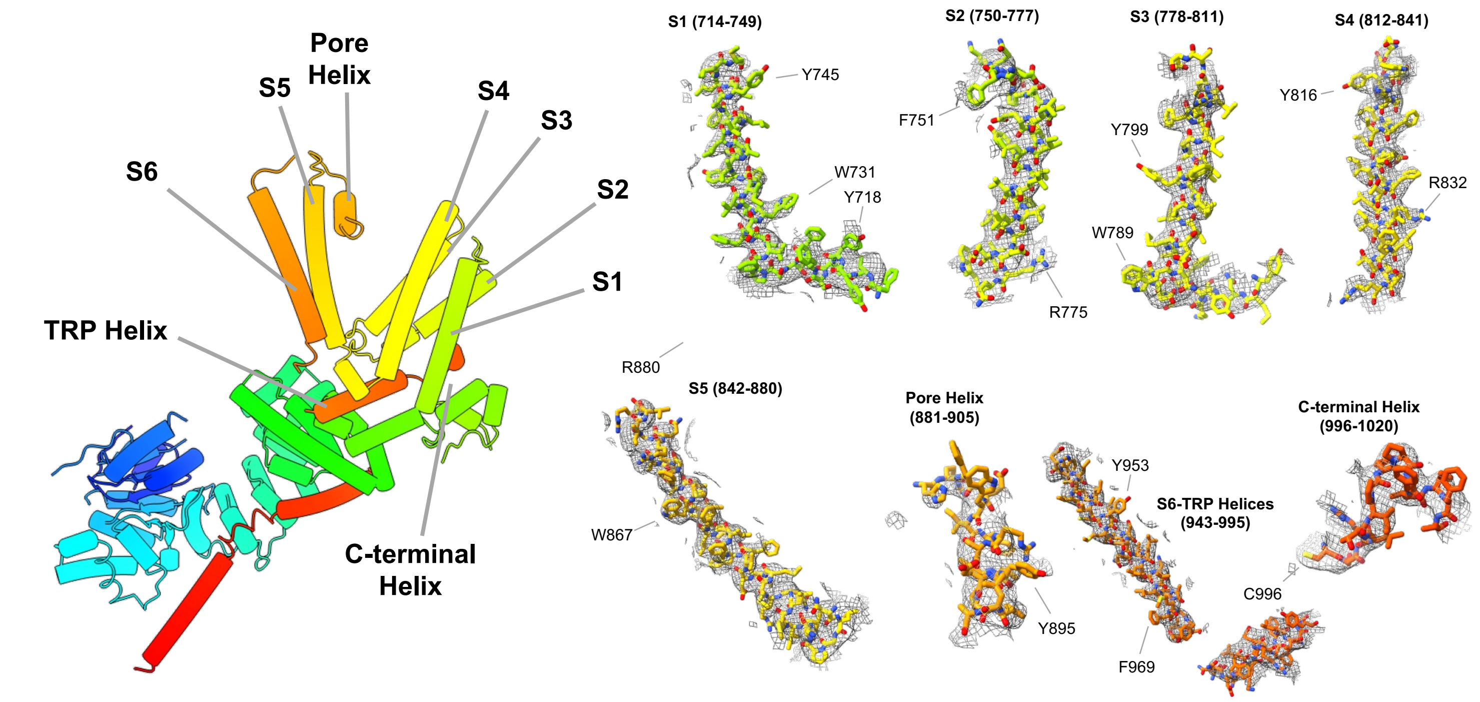

d *P. Major* TRPM8 determined in cell vesicles – menthol bound in the absence of calcium – open/semi-swapped – PDB: 9ZEZ (EMD-74123)

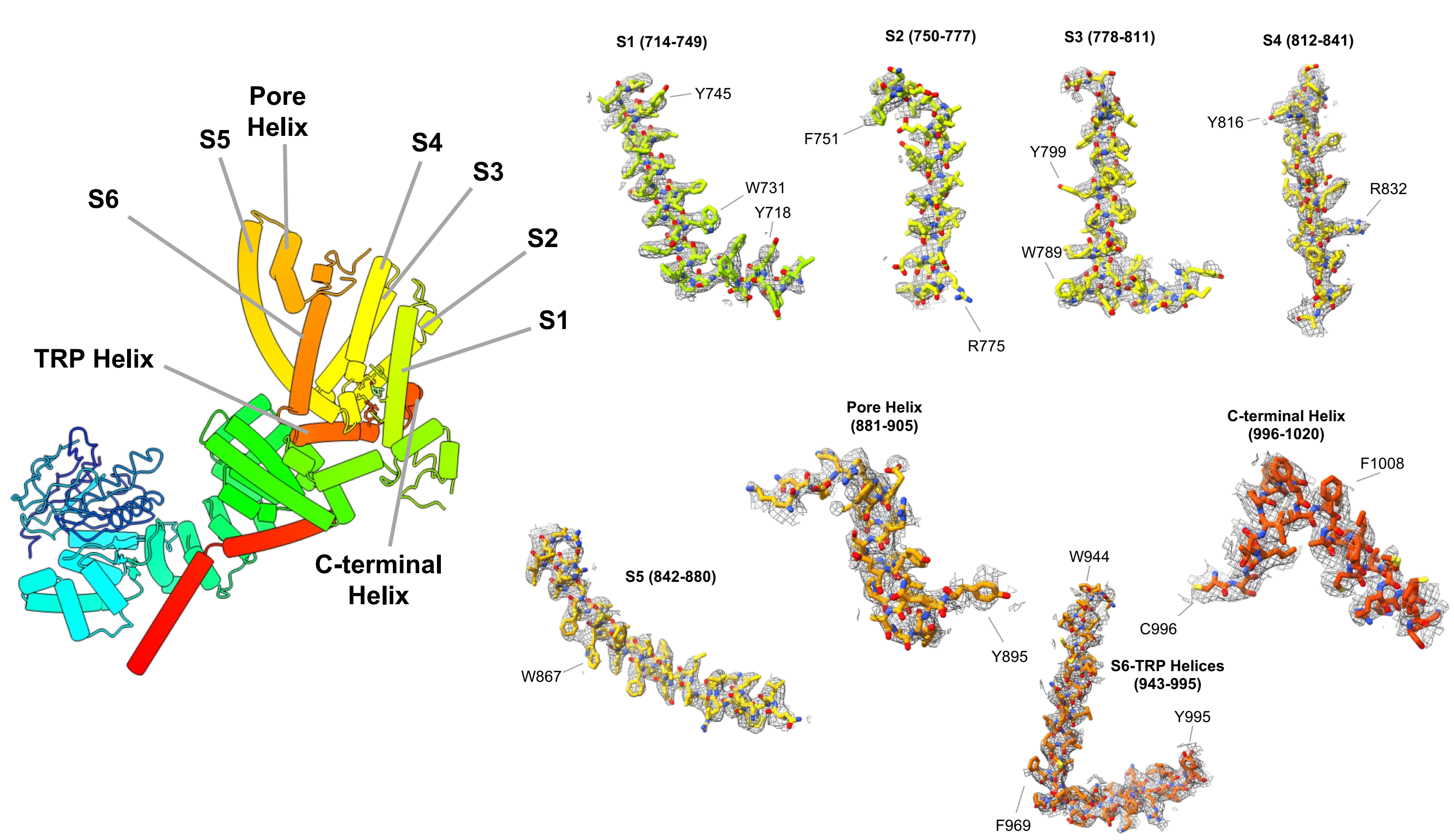

Supplementary Information Fig. 10 | Model and density representations. Zoned densities of transmembrane structure elements modelled in PDBs included in this study.

e *P. Major* TRPM8 determined in GDN – cold with high pH in the presence of calcium – open/semi-swapped – PDB: 9PAR (EMD-71444)

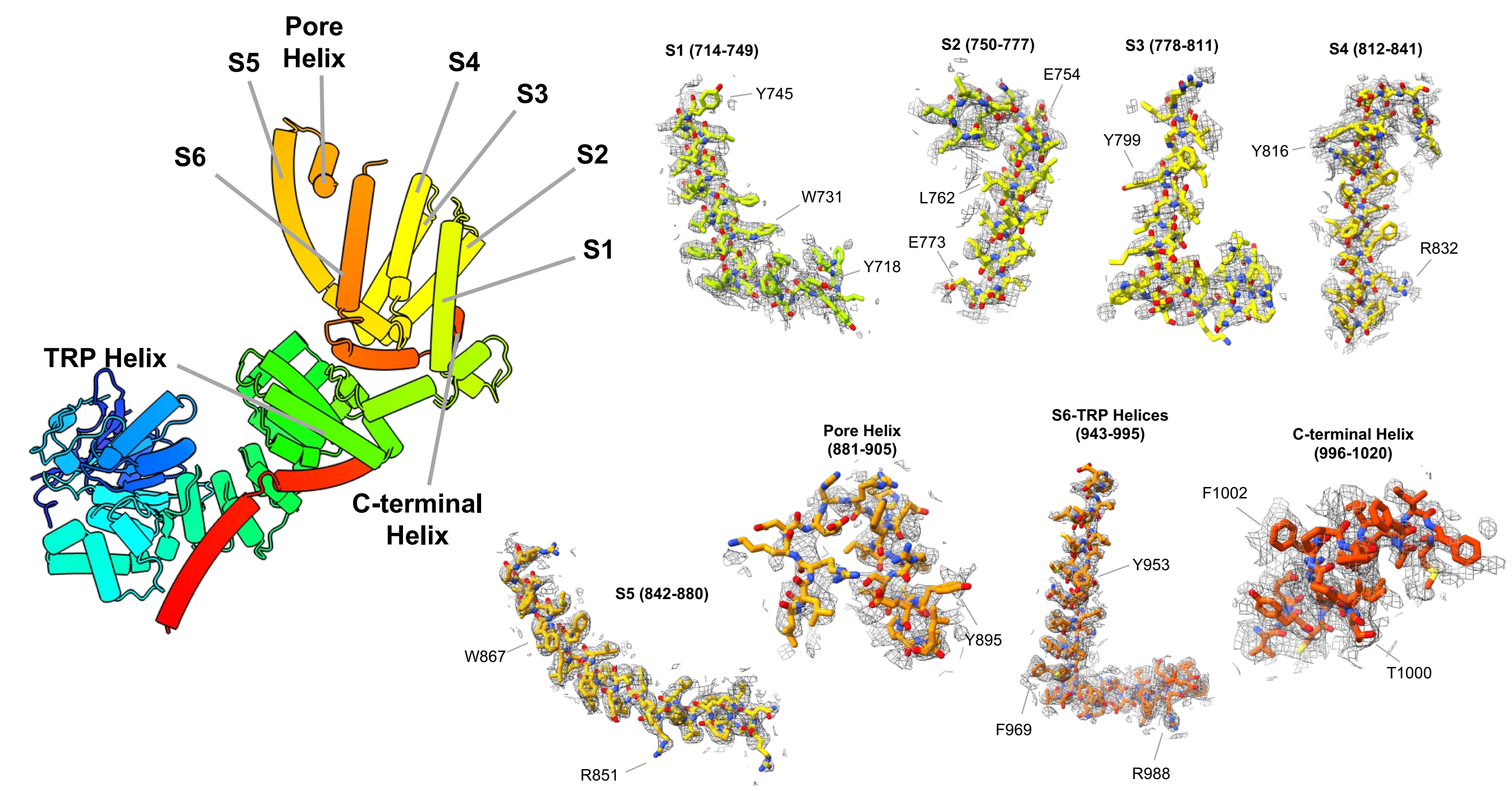

f Human TRPM8 determined in GDN – cold adapted in the absence of calcium – closed/fully-swapped – PDB: 9P8Y (EMD-71391)

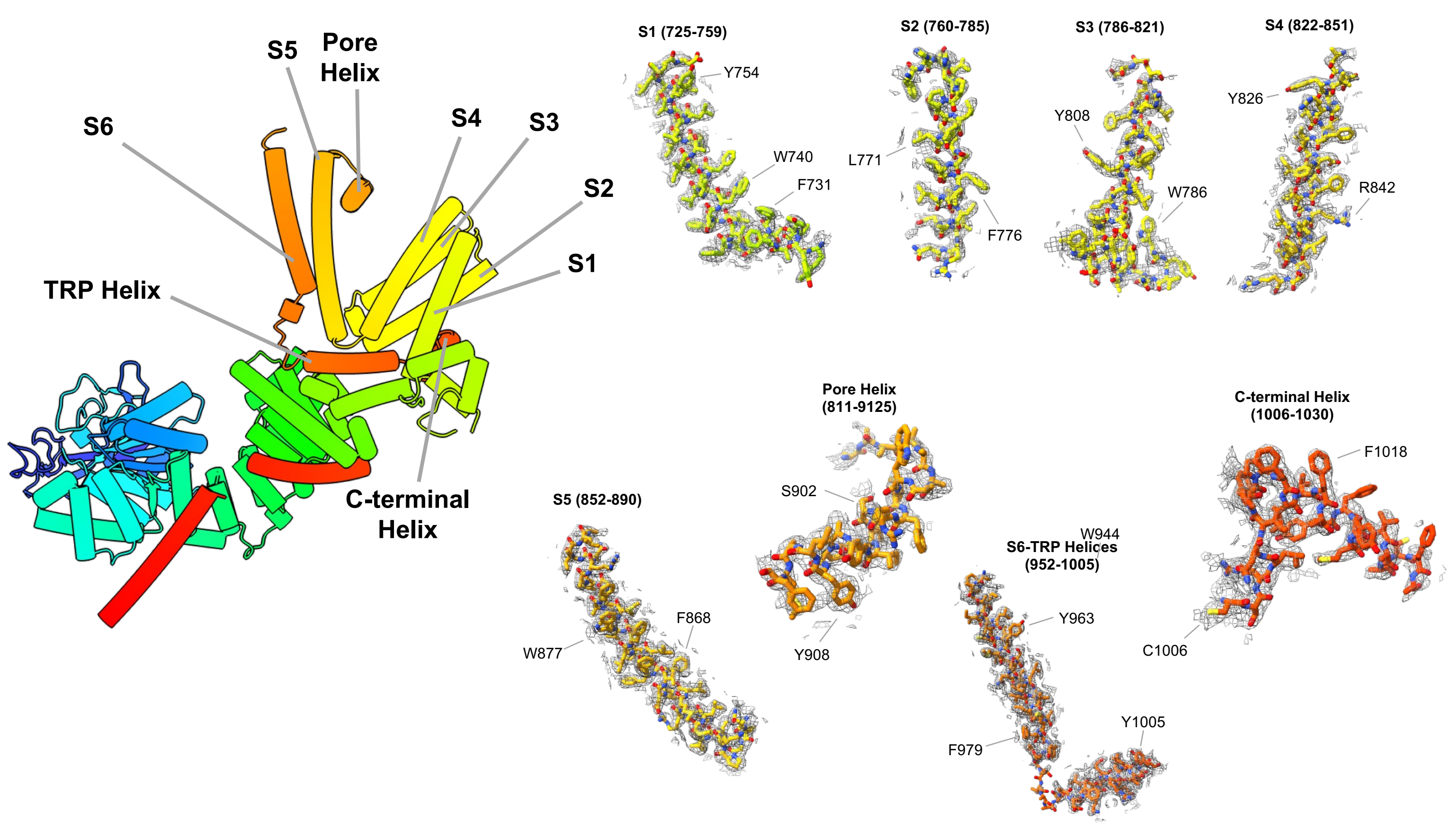

Supplementary Information Fig. 11 | Model and density representations. Zoned densities of transmembrane structure elements modelled in PDBs included in this study.

g Human TRPM8 determined in cell vesicles – cold in the absence of calcium – open/fully-swapped – PDB: 9ZF0 (EMD-74124)

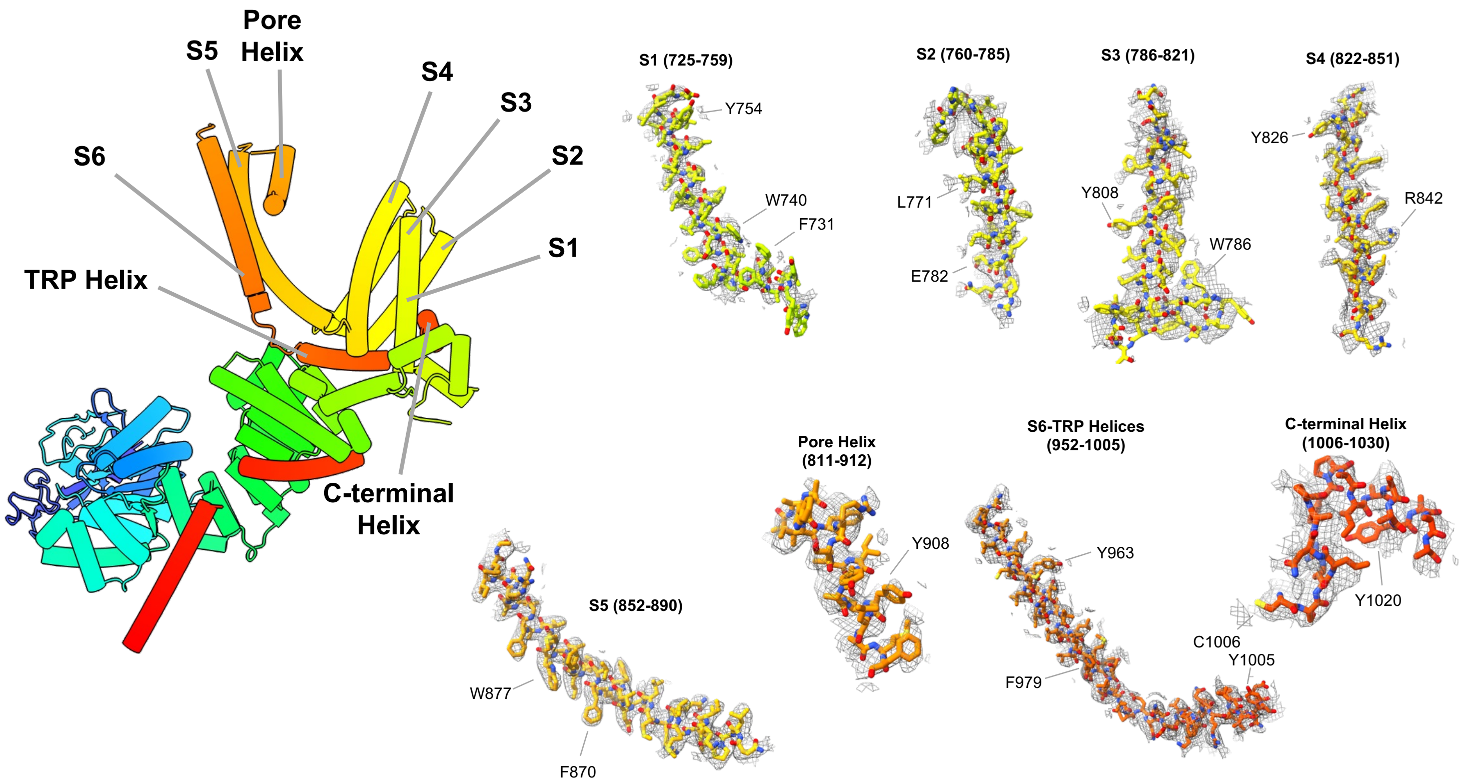

h Human TRPM8 determined in cell vesicles – menthol bound in the absence of calcium – open/fully-swapped – PDB: 9PB5 (EMD-71454)

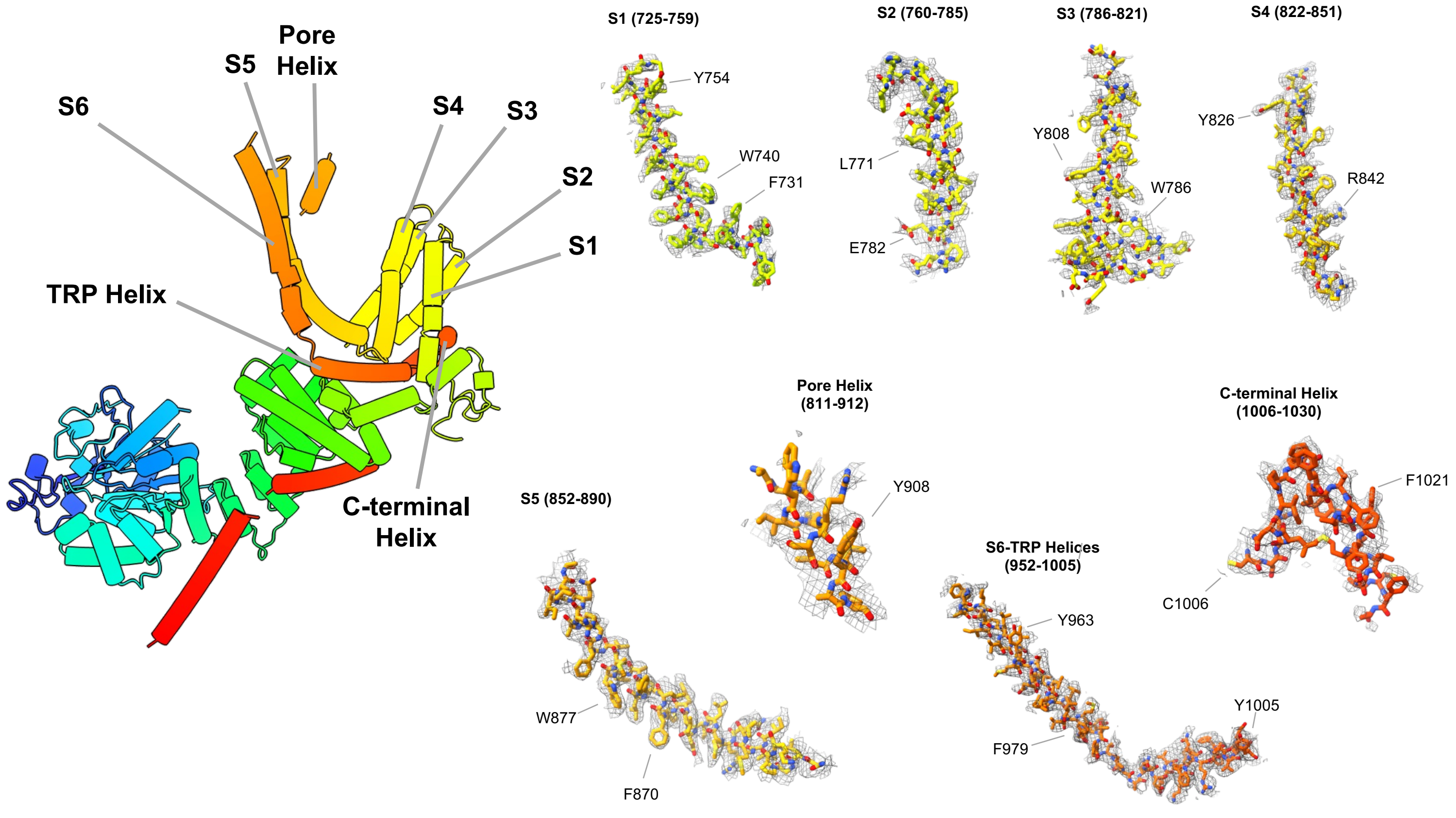

Supplementary Information Fig. 12 | Model and density representations. Zoned densities of transmembrane structure elements modelled in PDBs included in this study.

Unprocessed gel (Supplementary Fig. 1)

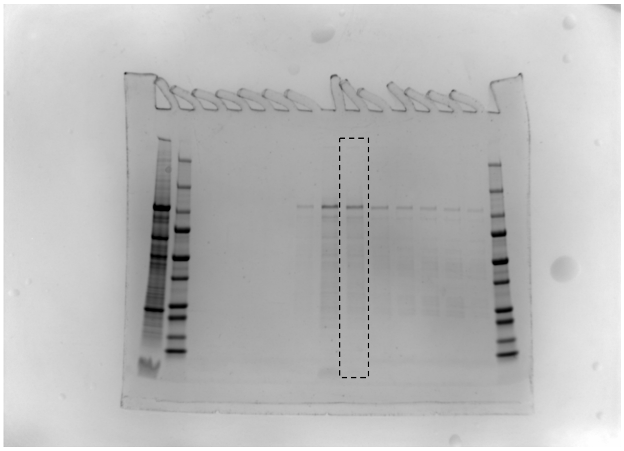

Supplementary Information Figure 1a

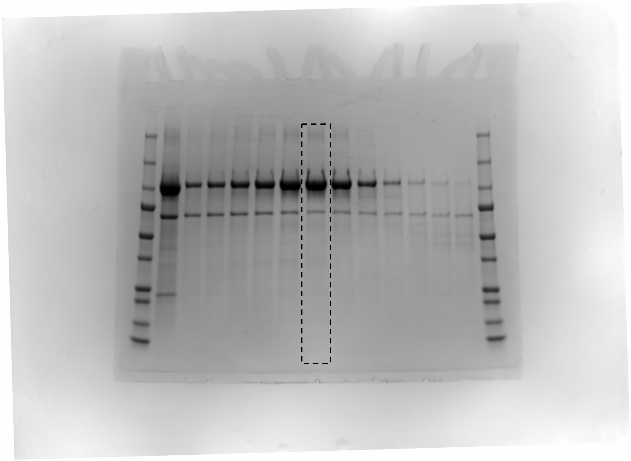

Supplementary Information Figure 1d

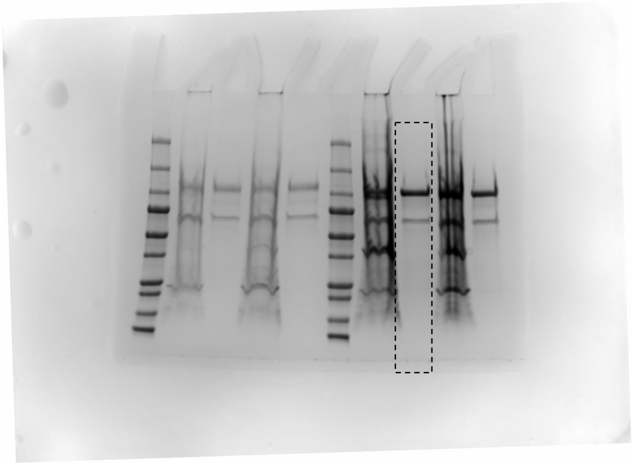

Supplementary Information Figure 1d

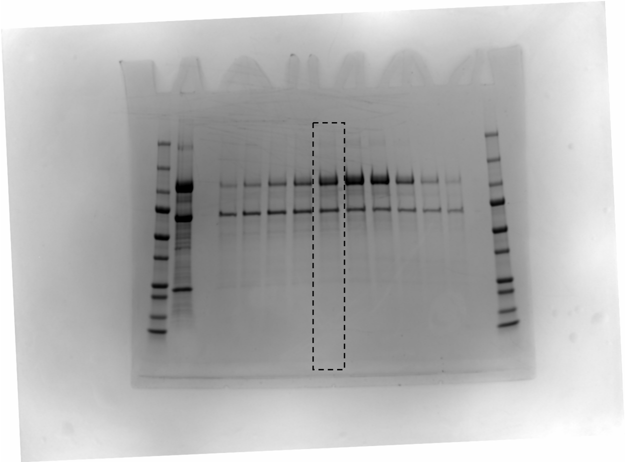

Supplementary Information Figure 1d

Supplementary Information Fig. 13 | Unprocessed SDS-PAGE gels. Uncropped gels shown in Supplementary Information Fig. 1. Blue rectangle denotes region of gel represented in Supplementary Information Fig. 1.
